# Coevolutionary aesthetics in human and biotic artworlds

**DOI:** 10.1007/s10539-013-9389-8

**Published:** 2013-07-06

**Authors:** Richard O. Prum

**Affiliations:** Department of Ecology and Evolutionary Biology, and Peabody Museum of Natural History, Yale University, New Haven, CT USA

**Keywords:** Aesthetic evolution, Mate choice, Art, Aesthetics of nature, Ornament, Aposematism

## Abstract

This work proposes a coevolutionary theory of aesthetics that encompasses both biotic and human arts. Anthropocentric perspectives in aesthetics prevent the recognition of the ontological complexity of the aesthetics of nature, and the aesthetic agency of many non-human organisms. The process of evaluative coevolution is shared by all biotic advertisements. I propose that art consists of a form of communication that coevolves with its own evaluation. Art and art history are population phenomena. I expand Arthur Danto’s *Artworld* concept to any aesthetic population of producers and evaluators. Current concepts of art cannot exclusively circumscribe the human arts from many forms of non-human biotic art. Without assuming an arbitrarily anthropocentric perspective, any concept of art will need to engage with biodiversity, and either recognize many instances of biotic advertisements as art, or exclude some instances of human art. Coevolutionary aesthetic theory provides a heuristic account of aesthetic change in both human and biotic artworlds, including the coevolutionary origin of aesthetic properties and aesthetic value within artworlds. Restructuring aesthetics, art criticism, and art history without human beings at the organizing centers of these disciplines stimulate new progress in our understanding of art, and the unique human contributions to aesthetics and aesthetic diversity.

## Introduction

The ontological diversity of art, the diversity of aesthetic properties, and the complexity of aesthetic change present enormous intellectual challenges to the philosophy of aesthetics, art criticism, and art history. Evolutionary biology shares similar challenges in the study of the diversity the earth’s organisms, their form and functions, the reconstruction of their evolutionary histories, and in understanding the mechanisms of evolutionary change. Neither evolutionary process nor aesthetic change is governed by deterministic laws (Beatty 2006; Sober 2006). Consequently, evolutionary biology must be investigated and understood in specific historical, taxonomic, comparative, developmental, and population contexts. But what of aesthetics? Are there more fundamental and substantive similarities between aesthetic change and organic evolution?

The possibility of substantive intellectual connections between aesthetics and evolutionary biology date back at least to Darwin’s (1871) *Descent of Man and Selection in Relation to Sex,* in which he elaborated the theory of sexual selection by mate choice. Darwin hypothesized that a female “aesthetic faculty”—literally an evolved “taste for the beautiful” that is exercised during mate choice—constitutes a distinct evolutionary force that leads to the evolution of ornamental traits in animals. Darwin used aesthetic language to describe mating preferences in order to communicate his hypothesis that animals were making choices on the basis of their own subjective sensory and cognitive experiences, and that these choices could lead to the evolution of arbitrarily attractive traits that are unrelated to any naturally selected advantage to the individual making the choice (Cronin 1991; Prum 2012). In the nineteenth century, Alfred Russel Wallace et al. aimed ferocious criticism at Darwin’s aesthetic theory of mate choice, arguing that any such process would be “neutralised” by the strong force of natural selection (Wallace 1889, 1895). Consequently, sexual selection was largely abandoned for nearly a century. Interestingly, the resurgent, contemporary version of sexual selection theory is largely organized around the Neo–Wallacean assumption that the evolution of mating preferences will be dictated by strong natural selection for honest indicators of male quality or mating efficiency, and does not follow Darwin’s own arbitrary, aesthetic view (Cronin 1991; Prum 2010, 2012). For this reason, the adaptationist trend in current sexual selection theory may have discouraged the development of productive connections to aesthetics. Following Darwin’s view, I have argued that the coevolution of display traits and mating preferences constitutes a distinct mode of *aesthetic evolution* in which the subjective sensory and cognitive experiences of mate choice can have evolutionary consequences independent of natural selection (Prum 2012).

Recently, Dutton (2009) has applied the adaptive, honest signaling sexual selection paradigm to the origin of art in people, and proposed that the evolution of the human capacity to produce art evolved through female mate choice for adaptive indicators of male mate quality or condition. These evolutionary psychology approaches to human aesthetics do little to explain actual variations in the form and content of human art. More recently, Davies (2012) has presented a more nuanced aesthetic analysis of the origins of human art. Here, however, I am concerned with broader commonalities between certain modes of biological evolution and the ongoing, historical process of aesthetic change in the human arts.

Given the ontological diversity of the human entities, artifacts, and performances that are generally considered as aesthetic, the fundamental goals of aesthetics are to establish accounts of the nature of aesthetic entities, aesthetic properties, aesthetic judgments, and the process of aesthetic change over time. Currently, the field of aesthetics has achieved little consensus on either a general theoretical framework, or specific solutions to these philosophical challenges. Here, I propose a coevolutionary aesthetic theory that provides new solutions to classic questions in biological and human aesthetics.

In this article, I explore the idea that art arises through a generalizable aesthetic process that is not exclusive to human beings. I propose that a coevolutionary aesthetic theory provides a heuristic, non-reductive account of the nature of art, the origin of aesthetic properties, and the process of aesthetic change. My proposal contradicts the assertion that the realm of aesthetics consists of an unnatural assemblage of phenomena invented by early modern philosophers (Carroll 2008). The lack of a coherent aesthetic theory by the ancients is not evidence that aesthetics is a modern invention. Rather, the existence of a distinct class of aesthetic phenomena is a modern, empirical discovery, which implies that there are additional aesthetic discoveries to be made. I propose that the diverse natural phenomena categorized as aesthetic entities arise through a common, intellectually discoverable aesthetic process.

Here, I present the core proposal of a coevolutionary aesthetic theory. I begin with an analysis of the diversity of aesthetic components of Nature. I argue that many biotic advertisements (e.g. animal courtship displays, fruits, flowers, etc.) share with human art a common mechanism of coevolving with their evaluations. Coevolution is the process of descent with reciprocal modification among entities through repeated dynamic interactions between them. I provide a brief description of sexual selection by mate choice as an example of a biological theory of evaluative coevolution. I then present a coevolutionary account of art. Specifically, I propose that art consists of a form of communication that coevolves with its evaluation (i.e. evolves in relation with, and in response to, its evaluation). I argue that many previously proposed anthropocentric requirements for art fail to exclude various non-human biotic advertisements. As Darwin (1871) proposed, I conclude that many animals share with humans the capacity for aesthetic agency—the state of participating in process of aesthetic expression, evaluation, judgment, and change.

Accordingly, many forms of biotic advertisements constitute non-human forms of art, which I call biotic art. Like biological evolution, art, aesthetic change and art history are population phenomenon. Accordingly, I expand on Danto’s (1964) concept of *The Artworld* to refer to any population of coevolving aesthetic entities and evaluators. The existence of myriads of non-human biotic artworlds constitutes exactly the type of challenge to aesthetic theory that Danto supposed in *The Artworld* (see epigraph).

My goal is a universal aesthetic theory that neither reduces, replaces, nor explains away traditional aesthetic inquiry with adaptationist, biological analysis. Rather, I propose that by focusing on the historical process of the coevolutionary entrainment of aesthetic producers and evaluators within aesthetic populations, we can provide broadly heuristic tools for understanding a diversity of issues in evolutionary biology, human aesthetics, art, and art history. Art and aesthetics are emergent consequences of advertisement communication, evaluation, choice, and evolutionary feedback; they cannot be reduced to more fundamental processes.

## Aesthetics of nature

Contemporary philosophical views of the aesthetics of nature are insufficient to comprehend the breadth of aesthetic complexity in nature. There are at least two important deficiencies—(1) absence of consideration of the potential aesthetic agency of non-human animals, and (2) an absence of consideration of the ontological diversity of natural phenomena that have become the objects of human aesthetic regard.

In previous aesthetic literature, the “aesthetics of nature” refers exclusively to human aesthetic experience of nature without consideration of the possibility of aesthetic agency of non-human organisms. There are several biological reasons to suspect that this view is overly narrow. First, there is nothing special about the sensory systems of humans to support our current privileged position in aesthetics. Humans are only one of millions of species of animals with elaborate and integrated sensory systems and derived communication signals that evolve by appealing to those senses. Human sensory systems are evolutionarily homologous with other vertebrates, and are mostly little changed from our mammalian and primate ancestors. In every sensory modality we have, human are greatly exceeded by the sensory complexity of at least some other vertebrates. Thus, there is no biological reason to assume that non-human organisms lack the sensory capacity for aesthetic experience. Sensory biology provides no legitimate reason to exclude the sensory experiences of non-human organisms from aesthetic consideration.

If non-human animals lack aesthetic agency—that is, the capacity to exhibit autonomous behavioral preferences based on cognitive evaluation of sensory information—then they must lack something other than the requisite sensory complexity. Is that something sensory judgment? Biologically, this is unlikely. Volumes of scientific literature document that many animals make numerous sensory evaluations or judgments as part of their lives—for example, among potential mates, fruits, or flowers (e.g. Ryan et al. 2009). Thus, as Darwin (1871) asserted, we have no reason to discount the potential for aesthetic agency by non-human animals.

To understand aesthetic process broadly, it is necessary to recognize the ontological diversity of the distinct, historically and mechanistically heterogeneous components of nature. Most philosophers have viewed nature as an aesthetically ‘flat’ tableau. Physical phenomena, land forms, geological objects, ecological communities, single species, individual organisms, and their performances are all treated as ontologically undifferentiated objects of human aesthetic regard (Budd 2003).

The lack of recognition of the potential for aesthetic agency in non-human animals has obfuscated fundamental differences in the ontology of the various components of nature that have traditionally been the subject of aesthetic human regard. The most fundamental, aesthetically relevant ontological category in nature is the distinction between the abiotic and the biotic. It is essential to recognize that a starry night sky and the song of a Wood Thrush (*Hylocichla mustelina*) are profoundly different phenomena whose properties arise by entirely different mechanisms—i.e. abiotic optical physics alone, or functional organismal physiology resulting from millions of years of genetic and cultural evolution, respectively. This fundamental fact appears to have been previously unrecognized in aesthetics.

Following the abiotic/biotic distinction in nature, we must further differentiate among various biological phenomena. Why, for example, do philosophers frequently cite flowers as examples of natural beauty (e.g. Kant 1987), but not plant roots? A biological component of the answer lies in the fundamental differences between the functional substrates of these two distinct components of the phenotype of a flowering plant, and the mechanisms by which these plant parts have evolved for their functions. Roots function in absorbing water and nutrients from the soil, and in stabilizing the plant in the soil. The functions of a root can be described entirely by physical and physiological data. In contrast, the flower is an advertisement to animal pollinators that provides a physical structure to mediate the exchange of nectar for pollination transport services. Some parts of the flower function in the production of ovules and pollen for sexual reproduction, but the conspicuous components of the flower—including its fragrance, and the number, shape, and color of petals and sepals—function in advertising to, attracting, indeed enticing, animal pollinators. Unlike roots, these components of the flower function through the subjective sensory perceptions and cognitive evaluations of other organisms. An animal regards the flower, evaluates that experience, and then decides whether to feed on its nectar, or to reject it and proceed to evaluate another competing floral nectar source. As a consequence of cognitive evaluations and foraging decisions, pollinators will either aid the plant in achieving pollination or not, and thus determine its reproductive success. Unlike the roots, a complete description of the function of a flower requires understanding the subjective sensory experiences, subsequent cognitive states, and decisions of the populations of pollinators that observe the flower. The optimal floral design cannot determined by adaptation to physical laws, but by the frequency dependent perceptual evaluations and cognitive states of a dynamically evolving population of potential consumers within a diverse community of competing floral species. As expected, wind pollinated flowers, whose function can be described entirely by physical forces and physiological data (e.g. elms, oaks), have notably are flowers that devoid of obvious aesthetic qualities.

## Aesthetic coevolution in nature

Flowers are biotic advertisements that function in a marketplace of animal sensory experiences and choices. The cognitive functional substrate of the flower creates the opportunity for a distinct mode of evolutionary process that does not occur to a plant’s roots. The flower’s visual and olfactory displays do not merely accidentally correlate to and exploit the desires of pollinators. Rather, the form of the flower and the sensory systems and judgments of the pollinator have *coevolved* with one another. Flowers have evolved to attract the sensory and cognitive systems of pollinators with memorable stimulus associated with a valuable reward, and pollinator nervous systems have evolved to evaluate, differentiate, remember diverse flowers. Furthermore, foragers develop their own floral preferences in response to their individual experiences with floral diversity. Flowers diversify among plant species because they are differentially successful at influencing the sensory judgments and cognitive decisions of different pollinators.^1^ The diversity of floral form and fragrance documents the evolutionary history of myriads of innovations to entice animals to aid them in affecting gamete transfer among individuals. No such process has occurred in the evolution of the plant’s roots.

In general, coevolved biotic signals in nature function explicitly as advertisements to other organisms. These signals include advertisements of sexual availability, advertisements of desire (such as baby begging calls), advertisements of the availability of ecological resources like nectar, pollen, or fruit, and advertisements of danger like the venomous rattlesnake’s rattle or the bold markings of the noxious skunk. Biotic advertisements may function within a single species or among multiple species.

Biotic advertisements evolve by a distinct mechanism from other communication signals because they are subject to, and coevolve with, the sensory judgments and evaluations of other organisms. To function, all communication signals must coevolve with their receivers, but not all signals coevolve with subjective sensory/cognitive *evaluations*. For example, both avian alarm calls and songs communicate to conspecifics acoustically. Bird songs often function in mate choice through conspecific sensory evaluation. By evaluating mate advertisement songs, a female bird may decide on one mate or another. However, a bird does not evaluate the quality of an alarm call before it decides whether to respond to its warning. Unlike bird song, once an alarm call is recognized as an alarm, its acoustic form or content is not subject to sensory evaluation and judgment. Likewise, variation in aesthetic quality among different individual *Stop* signs has no bearing on their meaning or function; we don’t just stop for the pretty ones. The fundamental difference in the coevolution between signal and receiver in different avian vocal signals creates the striking differences in signal complexity. It is not an accident that bird songs are generally considered beautiful and that bird calls are not.

## Coevolution by sexual selection

Biotic advertisements are those components, productions, or performances of an organism that function through, indeed invite, sensory evaluation. They evolve in contexts in which competing individuals cannot succeed by use of force, and must therefore use sensory/cognitive persuasion to achieve ecological, social or sexual success. Coevolution by sensory evaluation is responsible for the form and diversity of biotic advertisements. Evolutionary biologists have focused extensively on understanding the coevolution of biotic advertisement in various contexts, including pollination and fruit dispersers, but an especially rich example comes from the theory of the sexual selection through mate choice.

Darwin (1871) proposed the mechanism of sexual selection by mate choice, in which individuals of one sex—usually females—choose individual mates of the other sex. Darwin proposed that mate choice will result in the evolution of elaborate, ornamental advertisement traits. Darwin explicitly proposed that mating preferences were *aesthetic* preferences (Cronin 1991; Prum 2012). Further, Darwin’s concept of sexual selection was explicitly coevolutionary; even without a clear concept of genetics, he hypothesized the male ornament and female aesthetic preferences advance together (Prum 2012). Although it was almost completely rejected for the next century, Darwin’s idea has yielded a richly developed contemporary, coevolutionary sexual selection theory (see Andersson 1994; Rice 2004; Kokko 2005; Fuller et al. 2005; Prum 2012).

Models of sexual selection by mate choice are classified by the mechanism of evolution of mating preferences (Kirkpatrick and Ryan 1991). The simplest, aesthetic null model of display trait and mating preference evolution is the Lande–Kirkpatrick mechanism (Lande 1981; Kirkpatrick 1982; Kirkpatrick and Ryan 1991; Prum 2010, 2012), which is the complete, contemporary version of the arbitrary, aesthetic sexual selection of Darwin (1871) and the runway mechanism of Fisher (1915, 1930). In this aesthetic, null mechanism of evolution by mate choice, genes for display traits and mating preferences become correlated with each other. Then, selection by mating preferences on display traits creates indirect selection on mating preferences themselves. Thus, mate choice results in the self-organizing, evolution of arbitrary preferences or, as Darwin wrote, “standards of beauty” in each population and species.

Alternatively, following Alfred Russel Wallace, mate preferences can be hypothesized to evolve by strong natural selection (Cronin 1991; Prum 2010, 2012). These mechanisms predict the evolution of displays that are robust indicators of mate quality or other direct benefits. These Neo–Wallacean mechanism predict much reduced diversification in trait and preferences among populations (Prum 1997, 2010, 2012).

The aesthetic null model of sexual selection is explicitly coevolutionary; display traits and preferences coevolve with one another. Adaptive mate choice models have usually been constructed to eliminate the possibility of coevolution (e.g. Grafen 1990), but this has been done merely to demonstrate the theoretical efficacy of natural selection alone in the absence of trait/preference genetic correlation. There is no reason to hypothesize that adaptive mate choice does not involve trait/preference coevolution. Furthermore, there is overwhelming evidence of the coevolution of display traits and mating preferences in nature (Prum 2012).

## The coevolution of art

The ontological diversity, the multiple sensory modalities utilized, and the ever changing nature of human art is mirrored by the diversity, sensory properties, and dynamic change of coevolved biotic advertisement of non-human organisms. I propose that these similarities arise from a common coevolutionary aesthetic process.

Put simply, *art consists of a form of communication that has coevolved with its evaluation.* 2 Aesthetic coevolution is the process by which performances or artifacts and their evaluations mutually transform each other over time through their history of interactions. Aesthetic coevolution proceeds by the feedback between innovations in communication and evaluation upon each other. Evaluation involves a cognitive estimation of the sensory perception or cognitive experience engendered by the stimulus or artifact. Cognitive evaluation may involve both variation in immediately accessible sensory data and secondary cognitive associations. Criteria for aesthetic judgment by each individual may be determined by genetic, environmental, learned, cultural, psychological, individual, or random factors. Aesthetic evaluation can involve a single or multiple sensory modalities and cognitive dimensions. Judgment by an individual may be influenced (e.g. heightened, exaggerated, inhibited, etc.) by simultaneous or serial comparison to previous individual aesthetic experiences, but such comparative judgment is not essential. Coevolutionary process proceeds by the differential success and feedback of aesthetic entities and evaluative preferences (by genetic and/or cultural mechanisms; see below).

The coevolutionary aesthetic account provides an heuristic description of the mechanism of aesthetic change in the arts. For example, W. A. Mozart’s musical innovations transformed the aesthetic judgments of audiences—i.e. their capacity to imagine what music could be and what form it could take. The transformation of aesthetic judgments among observers dynamically fed back upon the production of new musical compositions and performances by other composers and artists, and fostered the creation of additional aesthetic innovations in music.

This coevolutionary framework also encompasses the “low arts” such as commercial advertisement, kitsch, toys, games, popular music, graffiti, etc. The concept excludes language in general, in which form and meaning *are* coevolved but not through evaluation, but would include rhetoric, literature, poetry, and slang in which the specific form of linguistic expression *does* coevolve with its evaluation.^3^ This coevolutionary concept of art would exclude technologies that coevolve with their functions but are not forms of communication (e.g. hinges, cars, computers, etc.). Like plant roots, the performance of such technological entities are specific to their explicit, non-sensory functions: i.e. a hinges capacity to rotate stably, etc. But, the coevolutionary concept would include various applications of such technologies that function in communication and coevolve with sensory evaluation (e.g. like decorative furnishings, automotive styling, and computer user interfaces and games).

A coevolutionary aesthetic theory does not assume or require any notion or mechanism of “progress” in aesthetic change. Rather, as in mate choice, aesthetic change can proceed in arbitrary directions depending upon the intrinsic consequences of evaluative success of various communication signals in creating coevolutionary feedback. As in sexual selection, I hypothesize that additional extrinsic forces (e.g. natural selection, cultural influences, institutional control, etc.) can act on evaluation to drive aesthetic process in non-arbitrary directions or constrain change to create stasis.

The coevolutionary aesthetic theory does not privilege the role of aesthetic producers in aesthetic process or change. Evaluative innovations may have just as powerful an effect on aesthetic coevolution as producer innovations. Collectors/audiences/readers/critics may play as active a role in the process of aesthetic change as artists/authors/performers. Thus, coevolutionary aesthetics is compatible with literary and aesthetic theories that focus on the active role of the audience/reader in the interpretation of artworks.

## Aesthetics as an historical process

Arthur Danto developed an influential aesthetic theory through analyses of Andy Warhol’s *Brillo Boxes* and through other aesthetic thought experiments (Danto 1964, 1981, 1984, 1997, 2003). Danto’s aesthetic theory presaged several essential elements of this coevolutionary aesthetic theory. Danto argued that whether an entity constitutes art requires understanding its appropriate historical context; he maintained that Warhol’s *Brillo Boxes* would not have been regarded as art 50, 100, or 500 years prior to the creation and first exhibition of this revolutionary art work in 1964. The critical feature of historical context, Danto suggested, is whether observers have an appropriate “theory of art” to appreciate the entity as art. A “theory of art” is an observer cognitive structure or capacity that critically affects the outcome of the evaluation of an otherwise identical sensory experience. Danto’s thought experiments precisely identified the role of the evolution of observer aesthetic preferences over time in the genesis of art and aesthetic change, and they implicate an essential role for historical process in the determining what is art.

Danto, however, did not fully recognize the inherently coevolutionary feedback between the production of aesthetic entities and aesthetic judgments. In a fully coevolutionary account of Danto’s classic example, the novelty of *Brillo Boxes* transformed the aesthetic experiences of some observers (most notably Danto). These aesthetically transformed observers included artists, critics, collectors, etc. who interacted directly or indirectly with artists, creating coevolutionary feedback on subsequent aesthetic production that contributed to innovation in the production of new aesthetic entities, and further coevolution with subsequent aesthetic evaluation.

The coevolutionary account of art is consistent with Danto’s view of the historical component of evaluator aesthetic preferences, or “theories of art”, as well as subsequent historical definitions of art (Levinson 1979, 1989). But the fully coevolutionary theory provides a broader understanding of the role of history and population processes in aesthetic change, and not just on the transformation of everyday objects presented in a new context to become art. Furthermore, the coevolutionary theory also eliminates the circularity of some historically ‘recursive’ definitions of art (Levinson 1989) by providing a clear-cut criterion for recognizing each origin of art—the initiation of the process of evaluative coevolution.^4^

## Artworlds are aesthetic populations

Another forward-looking component of Danto’s (1964) aesthetics lies in his concept of *The Artworld*—the social and cultural context in which theories of art evolve. The fundamental intellectual discovery achieved by Danto’s Artworld concept is the recognition of the critical role of a *population* of interacting aesthetic entities, producers, and aesthetic observers in aesthetic process. Danto’s Artworld consists of interactive communities of artists, critics, curators, performers, dealers, collectors, and general audiences who, together, influence theories of art. Thus, coevolution is a population phenomenon. The process of aesthetic change necessarily takes place in the context of a population of aesthetic producers/entities and observers/evaluators. Art is a form of social behavior.

Rather than narrowing Danto’s concept of the artworld, as Dickie (1974) proposed in his institutional theory of art, I propose to broaden it. An artworld is any population (or actively interacting group of subpopulations) of coevolving aesthetic entities, producers, and evaluators. The diversity of human cultural groups in which art coevolves requires that we recognize that there are multiple human “artworlds” with different populations of interacting producers and observers. Human artworlds can be highly exclusive, or broad and interpenetrating. However, human artworlds are merely a subsample of the many, diverse *biotic artworlds* which share the same fundamental aesthetic process of evaluative coevolution. (For convenience, I will use the term biotic artworld to refer to non-human artworlds even though humans are obviously also organisms.) In addition, I broaden the artworld concept to include all mechanisms of evaluative coevolutionary process. Thus, the coevolutionary account encompasses and is entirely consistent with, but is not limited to, the social processes that are central to Dickie’s institutional theory of art.^5^

The commonalities shared by biotic and human artworlds are more than just a convenient analogy. Their shared attributes arise from a common aesthetic process of evaluative coevolution independently manifest in different populations. Thus, bird songs are not merely natural sounds that humans may experience aesthetically, but products of the coevolution of acoustic structure and form with the aesthetic judgments of other individual birds within independent avian artworlds. Like *Brillo Boxes*, the history of aesthetic change affects the efficacy of aesthetic signals within avian art worlds. For example, Derryberry (2007) conducted experiments on White-crowned Sparrows (*Zonotrichia leucophrys)* in which she played recordings of contemporaneous male songs and male songs recorded 24 years earlier to females and males at the same locality. The older songs elicited behavioral responses nearly half as efficiently as the contemporaneous songs. Thus, aesthetic change in the White-crowned Sparrows affects the form of the vocal signals, the content of vocal preferences, and degrades the efficacy of historic vocal signals.

Artworld participation distinguishes biotic art from other natural entities that are sometimes considered aesthetically but are not art. Floral shape, color, and scent are not beautiful in the same way as a sunset or the twinkling stars, because flowers are products of coevolving artworlds consisting of multiple competing plant species communicating to, and coevolving with, the sensory systems of multiple discerning, judgmental individuals of different species of pollinators. By contrast, the color of the sunset and the twinkling of the stars are determined by physical mechanisms that cannot coevolve with our evaluations of them. The sunset, uncut gemstones, pinecones, and the night sky are not art because they do not coevolve with sensory evaluations of them. Sunsets and plant roots are not participants in any aesthetic population. Any aesthetic properties these entities may have can only arise because the observers have previously coevolved aesthetic preferences through interactions with genuine art forms, and these observers bring these aesthetic preferences to, or project them on, additional sensory experiences outside of the context in which they coevolved. Thus, I hypothesize that sunsets are not beautiful to gorillas and chimpanzees even though they have the sensory systems that are virtually identical to our own, because these apes are not participants in any artworld in which they could have coevolved visual aesthetic preferences to project onto the sunset.

I propose that the recognition of a myriad of biotic artworlds constitutes the “discovery of new class of art”. Exactly as Danto (1964) supposed in the quote cited in the epigraph, this discovery should challenge aesthetic theory in the same way that a new body of scientific facts challenges a scientific theory.

## An aesthetic theory of art

Having embraced two fundamental insights from Danto—the historical/transformational nature of art, and the Artworld as the aesthetic population—it is important to outline how this proposal differs, even radically, from Danto’s complete view. Most fundamentally, *contra* Danto, I am proposing an explicitly *aesthetic theory* of art in which the concepts of art and aesthetics are necessarily interdependent. In other words, I am proposing a single, common account of both phenomena.

Previous aesthetic theories of art have been based upon the centrality of individual aesthetic experience to the definition of art. These theories have failed from the lack of a defensible account of the nature of aesthetic experience (Carroll 1999). Although nearly all discussions of aesthetic theories of art have only entertained this aesthetic experience definition, there is no reason why aesthetic theories of art need be framed in this way.

Here, I propose an aesthetic theory of art based on the existence of a distinct *aesthetic process*—the coevolution of art and its evaluation. Aesthetic experience is not merely any perceptually based sensibility; rather, aesthetic experience includes those perceptual sensibilities and cognitive responses that are shaped by evaluative coevolution with aesthetic entities, or art. Accordingly, there is no art without aesthetics, and no aesthetics without art.^6^ Both phenomena can be distinguished from other entities or experiences by their shared history of coevolutionary aesthetic process.

Unlike previous attempts define the aesthetic, the coevolutionary criterion provides an explicit account of aesthetic experience that applies in a diversity of contexts, including humans and non-human species. A revolutionary implication of this argument is that the nature of the aesthetic is as historically dynamic as the nature of art. Aesthetic sensibilities are not merely the static, hard-wired, biological, essentialist, positive components of sensory experience. As the history of art history proves, aesthetic responses *do* change because they are themselves continually shaped by coevolution with the aesthetic entities of their regard.

Accordingly, I consider the concept of “anaesthetic art” to be an oxymoron. Analyses that imply that revolutionary artworks, like Marcel Duchamp’s *Fountain,* are non-aesthetic or anti-aesthetic fail to recognize that the aesthetic itself is *transformed* by aesthetic process just like the criteria for what constitutes an artwork. The history of art is full of radical artworks that were initially rejected that but were later reevaluated as aesthetically successful and even beautiful, as standards of aesthetic evaluation coevolved with the form, content, or mode of expression. The aesthetic is not a static concept that lies outside the arts in the reductionist realm of sensory biology or cognitive science. Although this may seem disruptive to traditions of the discipline, the aesthetic needs to be understood as a dynamic concept that exists because of, and through, its coevolutionary interrelationship with art.

This concept of the aesthetic may be rejected by many as overly broad. However, I do not make this proposal to be contentious or fashionably ‘post-human’, but to provide a constructive framework for understanding the nature and diversity of aesthetic phenomena, including the human arts.

## Omission and commission

The inclusion of coevolved biotic advertisements as art could be considered by some an as “error of commission” by a coevolutionary aesthetic theory. However, many previously proposed, anthropocentric requirements for the concept of art result in either the inclusion of some non-human biotic phenomena as art, or the omission of some universally accepted human art forms from recognition as art. Once aesthetics seriously engages with biodiversity, it becomes difficult to establish a non-arbitrary account of art that will circumscribe all recognized forms of human art but not include forms of non-human communication.

For example, art could be restricted to artifacts produced by an individual (Levinson 2006, 26–37). This condition would exclude flowers or the sexually dimorphic plumage ornaments of birds as art since these are not artifactual products of an individual, but parts of the individual. Such a requirement, however, could not exclude organismal vocal performances, physical displays, or display constructions such as the bowers made by male bowerbirds (Ptilonorhynchidae) (Frith and Frith 2004). The artifactual requirement would also exclude hairstyles or certain performances in which the human body is itself presented as an artwork (e.g. Abramović et al. 2005). Without arbitrarily restricting an “individual” to be a human being only, the artifactuality requirement cannot exclude all biotic art.

Art can be conceived as consisting only of entities that have culturally determined form. However, aesthetic culture is not limited to humans. There are over 4,000 species of birds—including oscine songbirds, parrots, hummingbirds, and bellbirds (*Procnias)*—that learn their songs from other individuals of their species (Kroodsma 1981, 2005; Searcy and Nowicki 2005; Saranathan et al. 2007). In many bird species, these learned songs have discrete structural features (i.e. notes and phrases) that make it possible to identify which individuals have learned from whom, and to describe exhaustively the process of cultural evolution in song of an entire population. For example, Robert B. Payne and colleagues documented the history and pattern of cultural evolution in song of Indigo Buntings *Passerina cyanea* over two decades in two populations in Payne et al. (1988). Payne documented that learning errors and individual vocal innovations constitute cultural mutations that can be subsequently traced as markers of cultural descent through the songs of other individuals that learn them over time. These avian vocal innovations live on in a population longer than the individuals that create them. Cumulative cultural evolution in learned bird song ultimately gives rise to local song variations and regional dialects that the birds themselves distinguish and recognize. Similar processes occur but are less well understood in the songs of whales (Noad et al. 2000) and in the ornamentation of male bowerbird bowers with colorful objects (Diamond 1982; Frith and Beehler 1998). The origin of song learning and culture in oscine songbirds occurred tens of millions years ago, perhaps even predating the completion of the break up of Gondwanaland (Barker et al. 2004). Although human culture is possibly 100,000 years old, songbirds have been doing ‘aesthetic culture’ on a grand scale for tens of millions of years. Thus, a cultural requirement for art will not distinguish biotic art from human art.

Art could be required to be the product of an *aesthetic intention* by an individual or a group of individuals. Aesthetic intention consists of the production, presentation, or performance of an aesthetic entity or artifact with conscious regard for its potential for evaluation by another individual. I argue that an aesthetic intention requirement does not clearly exclude biotic phenomena as art. Our judgments about whether intention exists are based largely on our own subjective experiences of what it is like to be human. Generalizing from our own experience, we easily grant aesthetic intention to the Cro-Magnon cave painters of Lascaux. But, we hesitate to infer intention from the actions of non-human organisms because they are so different from us. However, our doubts about animal intention should not be used as evidence against its existence.

If intention to act is considered as a cognitive mental state, then scientific observations may be relevant to such an inquiry. While a plant cannot have a conscious intention to attract a pollinator to its flowers, neurobiological evidence is consistent with the interpretation of aesthetic intention in singing male songbirds. The patterns of neural activity in the brain of a male Zebra Finches (*Taeniopygia guttata*) are functionally distinct when a male is singing to himself from when that male is singing to a potential mate (Jarvis et al. 1998). When a solitary male sings without an evaluating audience, the neural circuits of the male’s posterior vocal pathway that are involved vocal motor control and the anterior vocal pathway involved in song learning and vocal self-monitoring are all physiologically active. When a male sings the same exact song to a female (i.e. a potential evaluator of his vocal performance), the brain regions involved in vocal motor control are active, but the anterior vocal pathway circuits are not. The experiments were also controlled for the mere presence of a second, non-evaluating individual; the male’s neural circuit activity is the same when he is singing in the presence of another male, who is not a potential evaluator of his song, as it is when singing alone (Jarvis et al. 1998).

These experiments indicate that the male songbird’s cognitive state during vocal performance is distinctly different when the male perceives that his song has the potential to be evaluated. Thus, a singing male bird does not merely reproduce his song in mechanical response to a physiological drive, like a biological music box. Rather, the cognitive state of the male songbird during identical vocal performances differs with the potential for aesthetic evaluation of his song. These data are consistent with the presence of aesthetic intention in singing male Zebra Finch, and likely other animals with complex display behavior. For example, the careful process of the arrangement of found objects—including feathers, snail shells, fruits, flowers, bones, colorful insects, caterpillar droppings, etc.—by male bowerbirds at their bowers shows strikingly specific decisions that indicate similar pattern of conscious regard for their aesthetic evaluation (Frith and Frith 2004). As Dutton (2009) states, “even found objects—pieces of driftwood and the like—are transformed into intentional objects by the process of selection and display”. Although Dutton’s cluster concept of art excludes non-human animals, his intentionality criterion does not.^7^

Art could be required to have meaning, or *embodied* meaning (Danto 1981, 1984, 1997). Meaning is any additional information communicated by an artwork to the observer other than its existence, its objective form, and its availability for evaluation. Like human art, however, biotic art can also encode extra-sensory meaning such as information about mate quality. For example, the carotenoid pigment molecules which male House Finches (*Carpodacus mexicanus*) use to create red plumage ornaments come from the diet. If these pigments are rare in the diet, then the redness of the plumage may encode information about the quality of male’s diet, and perhaps the males overall genetic quality or health. Variation in such display traits could communicate an explicit, embodied meaning—male diet quality and condition—to a female with coevolved mating preferences.^8^ Similarly, warning coloration of venomous snakes or toxic butterflies have coevolved to mean that they are noxious and to be avoided. Thus, a requirement for meaning will not exclude all biotic advertisements as art. Furthermore, the requirement for meaning in an artwork would exclude various human art forms that have universally recognized aesthetic qualities, such as many forms of abstract music.

In conclusion, a full consideration of the complexity of biodiversity indicates that the necessary conditions of many contemporary concepts of art would lead to the inclusion of some biotic phenomena as art, or inappropriately exclude human phenomena that are nearly universally recognized as art. If inclusion of some form of biotic art is necessary to any appropriate account of the human arts, then the commission of biotic art by the coevolutionary theory is acceptable and appropriate. Art and the aesthetic are emergent properties of communication, sensory experience, cognitive evaluation, and coevolutionary feedback. We should be fully prepared for these properties to have emerged multiple times in the history of life.

In addition to biotic art, the coevolutionary account would include such human artifacts as commercial advertisements and packaging, and even pornography as art. Although the classification of these genres as art may be troublesome to some, it is not clear exactly how such genres are actually different from art. They may very well be bad art, but aesthetic failure does not constitute a failure to be art (Zangwill 2001). Indeed, Danto (2003) acknowledged that the original Brillo box was designed by the failed abstract expressionist painter James Harvey. Thus, Warhol’s *Brillo Boxes* was a duplicate of an everyday piece of commercial art whose design was informed by abstract expressionism. Furthermore, the boundaries between pornography and high art have been actively negotiated and dynamically changing for centuries. Many works that were once considered scandalous and pornographic are now canonical—such as Édouard Manet’s 1865 painting *Olympia.* Indeed, the 25,000 year old *Venus of Willendorf*—among the oldest known human artworks—could easily be considered pornographic given its pendulous breasts and explicit genitalia.

## Genetic and cultural coevolution

The specific mechanisms of coevolutionary feedback between aesthetic entities and evaluations in human and biotic artworlds are clearly very diverse. Yet, just as the ontological diversity of art itself does not prevent the recognition of the discipline of aesthetics (Davies 2003), the inherent diversity of coevolutionary aesthetic mechanisms should not prevent us from recognizing the results of these processes as art. Variation in the coevolutionary mechanisms of biotic and human art should be conceptualized as a product of coevolutionary aesthetic process acting on genetic, environmental, and cultural components of variation. Coevolutionary processes in biotic and human arts form a continuum of quantitative differences.

In the predominantly genetic case, evolution of genes for sensory preferences feeds back upon genes for production of aesthetic stimuli. In the predominantly cultural case, evaluative judgment feeds back on production or performance of new aesthetic entities through more diffuse mechanisms of learning, cultural evolution, other aesthetic influence, etc. I do not want to obfuscate the fundamental differences between genetic and cultural mechanisms of evolution. In many contexts, it is essential to differentiate between them in detail (e.g. evolution of bird song). However, regardless of the details of the mechanism that creates the historical entrainment between a stimulus and its evaluation, it is *coevolutionary* feedback between them that generates aesthetic change.

For example, the form and scent of a wild rose are determined by genetic factors (i.e. genes for the production of petals and odor molecules), by environmental factors (i.e. the availability of sun, soil, nutrients, water), and by individual variations (i.e. developmental accidents, disease). The form and content of a painting are partly determined in the same way by the product of genetic factors (i.e. genetic contributions to the artist’s body, sensory and cognitive systems), and other environmental factors (e.g. availability of pigment technologies, materials, etc.), and random individual effects (e.g. chance events, accidents, etc.). Further, a painting’s form and content are also determined by other cultural factors, which are an additional component of environmental variation (e.g. training, technique, stylistic norms coevolved with an artworld, etc.), and unique individual factors (i.e. the individual components of style, aesthetic innovation, psychological state, accidents). The differences in the mechanisms by which these two aesthetic entities achieved their form are not important to the consideration of whether they should be considered as artworks. Rather, the commonalities in the coevolutionary process shared between biotic and human artworlds establish their mutual status as art.

Even variation in human aesthetics production and evaluation has clear genetic components. We would all be comfortable with the conclusion that the artwork of a red-green color deficient painter could be influenced by the genetic variations in visual pigments that result in his seeing colors differently from most other artists and observers. The same would be true for a color blind art critic.

Thus, variation in human aesthetic process *does* have genetic components, and variations in some biotic art, such as some bird songs, have elaborate cultural components. We cannot characterize the mechanisms of aesthetic change in biotic and human art as categorically different. Biotic and human artworlds differ quantitatively, not qualitatively, in the contribution of genetic or cultural mechanisms to aesthetic coevolutionary process.

Coevolutionary aesthetic theory introduces a new component to the ontological diversity of aesthetic entities—the relative genetic or cultural basis of coevolutionary aesthetic process. This new conceptual challenge is actually no greater in magnitude than the well appreciated and accepted diversity in the ontology of current human art (Davies 2003). Aesthetic philosophy accepts, indeed *celebrates*, the ontological diversity of aesthetic entities as a documentation of the intellectual breadth, relevance, and importance of the discipline. Relative to the ontological extravagance of human aesthetic entities, the addition of biotic artworlds is a rather simple, albeit enormous, conceptual addition to aesthetics.

## Coevolutionary origin of aesthetic properties

The proposed coevolutionary account of art provides insights into a broad array of traditional and new issues in aesthetics. For example, coevolutionary aesthetic theory implies that the nature of the aesthetic properties elicited by an aesthetic entity—an object, part of an organism, artifact, or performance—arise through the history of coevolutionary interactions between aesthetic stimuli and evaluators within an artworld. Aesthetic properties can originate independently in independent artworlds. Thus, coevolutionary aesthetic theory is inconsistent with the formalist proposal that aesthetic properties and values are inherent in the form or properties of an aesthetic entity (Zangwill 2003). Likewise, aesthetic properties do not arise solely in the aesthetic experience of the observer, as in earlier aesthetic theories of art (Carroll 2008). Rather, aesthetic properties consist of different modes and qualities of interaction between an aesthetic entity and its evaluation. Aesthetic properties arise through coevolutionary aesthetic *process* within an art world. As Walton (1993) wrote, “Aesthetic pleasure is not just pleasure in my admiration of something, but it is getting me to admire it”.

In the broadest sense, beauty is a positive aesthetic property that coevolves through positive sensory engagement of an evaluator. Thus, an aesthetic stimulus is beautiful if it engages the receiver within its own artworld through a positive, coevolved aesthetic experience. By positive, I mean that an aesthetic experience of beauty leads to associational behavior—the desire for continued association with the stimulus that produces that experience. In this sense, the aesthetic experience of beauty is not detached nor disinterested as Kant (1987) proposed in *Critique of Judgment*, but actually requires, and coevolves by, the invitation and maintenance of evaluative engagement. Beauty differs most fundamentally from other aesthetic properties in the dominance of the sensory content of the aesthetic experience over other cognitive components (Zangwill 2001).

Common criticisms of the proposition that aesthetic can be informed by nature are that aesthetics is about much more than beauty, and that the aesthetics of nature consists merely of examples of beauty. Contrary to this notion, an entire class of aesthetic, biotic advertisements has evolved to create the opposite of the experience of beauty—the experience of coevolved ugliness. Such purposefully repellent aesthetic stimuli inspire aversion in evaluators—the desire to disengage with, and avoid the stimulus. Not all examples of ugliness have the same negative aesthetic value. The aesthetically repellant is distinguished from the incidentally ugly by its history of coevolution with aesthetic judgment.^9^ Humans and many other organisms have evolved innate revulsion to the odor of rotting flesh, possibly as an adaptation to avoid infection. But this revulsion is not aesthetic because no features of the process of bacterial decay have coevolved with our revulsion. Rotting flesh is merely repulsive, not aesthetically repulsive.

A vivid biotic example of coevolved aesthetic revulsion is the phenomenon of warning signals in venomous, toxic, and noxious organisms, called *aposematism* (Wickler 1968; Ruxton et al. 2004). Venomous coral snakes have evolved a striking pattern of brilliant red, yellow, and black bands to communicate to other organisms that they are dangerous and should not be molested. Birds of many species flee in fear from this colorfully contrasting stimulus. In some organisms, fear of these color patterns is an innate, coevolved response. For example, naïve young chickens are innately afraid of artificial coral snake color patterns (Ruxton et al. 2004, 249). In some species, aversion to an aposematic signal is learned. The vivid color patterns and the aversion to them evolve because organisms that evaluate the brilliant patterns as repellant have a higher chance of surviving, and snakes that display these brilliant and memorable color patterns avoid the risks of engagement. Other aposematic color advertisements include the black-and-yellow stripes of bees and wasps, the red-and-black pattern of the toxic monarch butterfly, the brilliant colors of the toxic poison-dart frogs, the bold patterns of noxious skunks, etc. The rattlesnake’s rattle is an unusual example of an aposematic acoustic advertisement—a coevolved, repellant *acoustic* display.^10^

Because aesthetic properties originate independently in independent artworlds, biotic examples of negative aesthetic value cannot be distinguished from beauty on the basis of their intrinsic form. Aposematic warning color patterns are similar to those used for mate attraction in some avian artworlds, and are commonly viewed as beautiful by humans. The rattlesnake warning rattle is acoustically similar to the whirring chirps that some crickets perform to attract mates. Similar aesthetic stimuli have coevolved distinctly opposite aesthetic properties in independent artworlds. Thus, positive or negative aesthetic value, or the valence of aesthetic experience, can only be recognized by understanding the coevolved aesthetic experiences of natural observers in those artworlds. The nature of the coevolutionary interaction within an artworld defines the aesthetic properties that result.

The coevolution of extra-sensory meaning encoded by aesthetic stimuli creates the opportunity for the evolution of more complex aesthetic properties including cute, elegant, garish, delicate, ironic, noble, heroic, kitschy, etc. These more complex aesthetic properties are contingent upon the existence of meaning in aesthetic experience, but beauty need not be. Beauty *can* communicate meaning—as in the possibility that red plumage can signal a bird’s diet and quality, or gold leaf the wealth of an artist’s patron. But beauty is not dependent upon meaning to exist. Beauty can be arbitrary in form. Thus, red plumage may lack meaning to other birds if the carotenoid pigments are abundant in the diet yet still be arbitrarily attractive. A common pigment could not provide mate quality information, even though the red plumage would still provide an identical sensory experience. A complete description of the aesthetic experience of red plumage would depend upon whether the red pigmentary color evolved under conditions of dietary pigment scarcity, and consequently encodes information about diet and mate quality, or under conditions of abundance and is merely beautiful in absence of encoded meaning. Likewise the presence of gold leaf in an artwork would not communicate investment and inherent value if gold were abundant and cheap, but it would still produce an identical sensory effect. Negative aesthetic values—coevolved repellence—are also contingent on meaning because simple repellence would not lead to coevolutionary entanglement that is necessary for aesthetic process to occur.

Because attraction and engagement are the most efficient contexts for the maintenance of coevolutionary process, beauty is the overwhelmingly predominant aesthetic property in both biotic and human aesthetics. Beauty is first and foremost among all aesthetic values. The predominance of beauty in the human and biotic arts is not accidental, nor is it the result of an anti-progressive, conservative, reactionary, institutional, or economic forces. Beauty is predominant because positive aesthetic engagement will foster the most persistent, detailed, and profound forms of the coevolutionary interactions that drive all aesthetic process. In other words, beauty is the null, or default, aesthetic property of art.

## Conclusion

The current discipline of aesthetics is organized exclusively around the aesthetic productions and aesthetic experiences of human beings. This narrow framework has prevented to recognition of generalizable the principles or solutions to philosophical problems of aesthetics.

I propose that the disciplines of aesthetics, art criticism, and art history should encompass both humans and non-human organisms, and that they should span evolutionary biology, behavioral biology, psychology, and the humanities. Unlike earlier sociobiology or current evolutionary psychology research programs, this interdisciplinary theory is *not* a reductionist campaign to explain away the humanities by appeal to the overwhelming (and simplistic) power of natural selection. Rather, this coevolutionary aesthetic theory is based on the understanding that the subjective, individual, sensory and cognitive experiences of humans *and* non-human animals can have (genetic or cultural) evolutionary consequences that are independent of, and not reducible to, natural selection.

Aesthetics is an emergent property of communication, sensory biology, cognitive evaluation, choice, and coevolutionary feedback. When these features arise, they cannot be reduced entirely to more fundamental mechanisms or processes. Coevolutionary aesthetic theory will provide a new, productive intellectual bridge between evolutionary biology and the humanities. Although coevolutionary aesthetic theory makes the radical proposal that many biotic advertisements are forms of art, it simultaneously provides an intellectual framework for understanding the nature of the aesthetic processes that give rise to human art forms.

In the history of cosmology, each step we humans have taken away from being the organizing center of the universe has produced an expansion in our knowledge of reality, and has enhanced human appreciation of our origins and uniqueness. Likewise, restructuring the disciplines of aesthetics, art criticism, and art history to remove human beings from their organizing centers will also foster progress in genuine understanding human art, and the unique human contributions to aesthetic diversity.

## Footnotes

1 If bees and other pollinators were not making real, cognitive choices based on their subjective experiences and individual preferences, then the diversity of flowers would not exist. If bees were merely responding reflexively to floral stimuli, then all flowers would converge on that singular form that would elicit apian foraging response with the greatest efficiency.

2 I am positively inclined to the proposal that the coevolutionary account may provide a full-fledged definition of art, but I will not attempt to defend that position here.

3 Further work would be required to distinguish at a fine scale which distinctive features of different languages are the result of evaluative coevolution. It may be that a greater component of linguistic change occurs within populations of speakers as the result of either conscious or unintentional coevolutionary response to evaluation; aesthetic fashion may play a larger role in language differentiation than I realize.

4 Levinson's (1979, 1989) proposed that art consists of any entity of a form historically recognized as art. Although it encorporates Danto's assertion of the historical nature or art, this criterion was appropriately criticized as circular because it merely begs the question of the origin of the historical criteria for recognizing art. The coevolutionary account avoids this failure.

5 Dickie's institutional theory can be understood as hypothesizing that art is shaped by coevolutionary feedback between artistic production and institutional approval or sponsorship. While this mechanistic hypothesis for aesthetic change is plausible, it does not characterize all mechanisms of aesthetic change which may coevolve with non-institutional forms of approval and evaluation (e.g. folk art, cave paintings, and, of course, biotic arts).

6 As proposed above, some aesthetic experiences can be solicited by sensory perceptions of non-art (like the sunset), but these aesthetic experiences are ultimately due to the existence of aesthetic preferences that coevolved with art in some other context that have been projected on to a perception of non-art.

7 In response to his perception of cultural bias, personal idiosyncracy, and misplaced concern for marginal examples within contemporary aesthetic accounts of the nature of art, Dutton (2009) has proposed the use of a list of “recognition criteria” for art, any combination of which can be used to recognize a work of art from non-art. Following on Wittgenstein and Weitz (1956), these “cluster concepts” of art have received significant criticism (Davies 2004). Dutton represents his perspective as “naturalistic” because it is based on direct observations of the diversity of art (which has already been limited to human examples). Dutton’s account fails, however, because it lacks any account of the *natural process* that gives rise to the aesthetic and ontological diversity of art. Dutton's recognition criteria are subject to the same potential cultural biases, personal idiosyncracies, and cherry-picked emphases as the previous definitions he rejects.

8 Meaning in biotic art arises through natural selection on aesthetic preference—i.e. the practical outcome of evaluation matters, but these coevolved signals will still be beautiful despite their practicality. This is functional aspect of natural beauty that was overlooked by Parsons and Carlson (2008).

9 We may ultimately require new terms to refer to coevolved beauty and ugliness, etc. to distinguish these proper aesthetic properties from those incidental sensory experiences that can elicit similar sensory evaluations—aesthetic projections if you will—which have not coevolved with these stimuli.

10 Within their biotic artworlds, these negative aesthetic values may share something in common with the negative aesthetic value that humans experience in revolting art, horror movies, or art forms of extreme suffering, including tragedy. Although these human art forms can engage human observers, this engagement requires the coevolution of preferences for the maintenance of engagement with explicitly repulsive or disturbing content.
